# Unacylated Ghrelin is associated with the isolated low HDL-cholesterol obese phenotype independently of insulin resistance and CRP level

**DOI:** 10.1186/1743-7075-9-17

**Published:** 2012-03-13

**Authors:** Juan-Patricio Nogueira, Marie Maraninchi, Sophie Béliard, Anne Marie Lorec, Bruno Berthet, Audrey Bégu-Le Corroller, Noémie Dubois, Rachel Grangeot, Catherine Mattei, Jean Gaudart, Alain Nicolay, Henri Portugal, Bernard Vialettes, René Valéro

**Affiliations:** 1 Aix-Marseille Univ, UMR 1260, 13005 Marseille, France; 2 UMR INSERM 939, La Pitié Hospital, Paris, France; 3 Aix-Marseille Univ, APHM, Sainte Marguerite Hospital, Department of Biochemistry, 13005 Marseille, France; 4 Aix-Marseille Univ, APHM, La Timone Hospital, Department of Digestive Surgery, 13005 Marseille, France; 5 Aix-Marseille Univ, APHM, La Timone Hospital, Department of Nutrition, Metabolic diseases, Endocrinology, 13005 Marseille, France; 6 Aix-Marseille Univ, EA 3283, Biostatistics Research Unit, Laboratory of Education and Research in Medical Information Processing (LERTIM), 13005 Marseille, France; 7 Service de Nutrition, Maladies métaboliques et Endocrinologie, Hôpital La Timone, 264 Rue Saint Pierre, 13005 Marseille, France

**Keywords:** Adiponectin, Apolipoprotein, Cardiovascular risk, Ghrelin, HDL-cholesterol, Inflammation, Insulin resistance, Lipids, Obesity

## Abstract

**Background:**

Low plasma high-density lipoprotein-cholesterol (HDL-c) level is commonly present in obesity and represents an independent cardiovascular risk factor. However, obese patients are a very heterogeneous population and the factors and mechanisms that contribute to low HDL-c remain unclear. The aim of this study was to investigate the association between plasma HDL-c levels and plasma hormonal profiles (insulin, adiponectin, resistin, leptin and ghrelin) in subsets of class II and III obese patients.

**Methods:**

Fasting plasma levels of glucose, total cholesterol, LDL-c, HDL-c, triglycerides, free fatty acids, apoproteins A-I, B-100, B-48, C-II, C-III, insulin, hs-CRP, adipocytokines (adiponectin, resistin, leptin), unacylated ghrelin, body composition (DXA) and resting energy expenditure were measured in three subsets of obese patients: 17 metabolically abnormal obese (MAO) with metabolic syndrome and the typical metabolic dyslipidaemia, 21 metabolically healthy obese (MHO) without metabolic syndrome and with a normal lipid profile, and 21 isolated low HDL-c obese patients (LHO) without metabolic syndrome, compared to 21 healthy lean control subjects.

**Results:**

Insulin resistance (HOMA-IR) increased gradually from MHO to LHO and from LHO to MAO patients (*p *< 0.05 between MHO and MAO and between LHO and MAO). In multiple regression analysis, serum unacylated ghrelin levels were only positively and independently associated with HDL-c levels in the LHO group (*p *= 0.032).

**Conclusions:**

These results suggest that, in class II and III obese patients with an isolated low HDL-c phenotype, unacylated ghrelin is positively associated with HDL-c level independently of insulin resistance and CRP levels, and may contribute to the highly prevalent low HDL-c level seen in obesity.

## Background

Obesity is becoming a global epidemic, and the prevalence of class II and III obesity is constantly increasing. Insulin resistance is common in obese patients and is a cornerstone in the pathophysiology of metabolic syndrome [1]. Cardiovascular diseases are the primary cause of morbidity and mortality in obesity. Each 5 kg/m^2 ^increase above a body mass index (BMI) of 25 kg/m^2 ^results in a 40% increase in cardiovascular mortality [2]. A dyslipidaemia is often present and represents a major cardiovascular risk factor. This typical dyslipidaemia is characterised by the quartet: hypertriglyceridaemia, reduction in HDL-cholesterol (HDL-c), increased small and dense low-density lipoproteins (LDL) and post-prandial hyperlipidaemia [3]. A number of studies have shown that low plasma HDL-c levels are an independent cardiovascular risk factor [4].

However, obese patients are a very heterogeneous population. For instance, the new concept of a metabolically healthy obese (MHO) group has emerged [5]. This subset of obese individuals, who could represent up to 30% of the obese population [6], is characterised by high insulin sensitivity, no sign of hypertension, normal lipid, inflammation and hormonal profiles and a low incidence of cardiovascular diseases [5]. Although still discussed, visceral adiposity appears to be more closely linked to insulin resistance, atherogenic dyslipidaemia, decreased blood adiponectin level and inflammation than subcutaneous adiposity [7,8]. Nevertheless, the criteria used to distinguish between MHO and at-risk obese individuals are poorly defined and the factors and mechanisms that could explain these different metabolic profiles, particularly HDL-c level, remain unclear.

Adipocytokines secreted by adipose tissue, such as adiponectin, leptin and resistin, play major roles in the regulation of energy metabolism and/or insulin sensitivity [9,10] and could directly or indirectly modulate lipid metabolism [11]. Likewise, the ghrelin hormone, produced predominantly by the stomach, is involved in energy balance regulation, insulin sensitivity and adiposity [12,13]. However, no study has yet investigated the link between HDL-c level and hormonal profiles in different subsets of obese patients.

In the present study, our main objective was to investigate the potential link between HDL-c levels and hormonal profiles (insulin, adipocytokines, ghrelin) in different subsets of obese patients: 1) the metabolically abnormal obese (MAO) with metabolic syndrome and the typical metabolic dyslipidaemia; 2) the metabolically healthy obese (MHO) with no metabolic syndrome and with a normal lipid profile; and 3) the low HDL-c obese (LHO) with no metabolic syndrome but with an isolated low HDL-c level; in comparison to 4) healthy and lean control subjects.

## Methods

### Subjects

This study was conducted in accordance with the Declaration of Helsinki, approved by the institutional ethics committee, and written informed consent was obtained from all patients. We recruited the obese patients from among those who were referred to our nutrition department with an indication for bariatric surgery (class II and III obesity). All obese patients and 21 lean normolipidaemic control subjects (5 males, 16 females; mean age: 33 ± 6 years (yr); mean BMI: 22.1 ± 2.9 kg/m^2^; waist circumference: 74.8 ± 7.6 cm) underwent a physical examination and laboratory tests to ensure they had no exclusion criteria. The patients were eligible if they did not smoke and drink less than 20 g alcohol/day, if they had normal fasting blood glucose (< 6.1 mmol/L), normal blood pressure (systolic blood pressure < 140 mmHg and diastolic blood pressure < 90 mmHg), no hepatic, renal, thyroid or haematological abnormalities, no inflammatory disease, and had not been treated with a drug that could interfere with insulin sensitivity or lipid metabolism. We included 59 obese patients who were divided into three groups: a first group of 17 obese patients with metabolic syndrome according to the National Cholesterol Education Program's Adult treatment panel III report [14] and determined by the presence of the three following criteria: central obesity (waist circumference > 102 cm for males and > 88 cm for females), hypertriglyceridaemia (triglycerides: ≥ 1.7 mmol/L) and low HDL-c (< 1 mmol/L for males and < 1.3 mmol/L for females) (MAO group) (6 males, 11 females; mean age: 38 ± 11 yr; mean BMI: 43.3 ± 4.7 kg/m^2^; waist circumference: 129.0 ± 14.3 cm); a second group of 21 obese patients with no metabolic syndrome and with normal HDL-c level (> 1 mmol/L for males and females) (MHO group) (4 males, 17 females; mean age: 37 ± 5 yr; mean BMI: 41.4 ± 4.1 kg/m^2^; waist circumference: 121.1 ± 13.5 cm); a third group of 21 obese patients with no metabolic syndrome but with an isolated low HDL-c level (< 1 mmol/L for males and females) (LHO group) (6 males, 15 females; mean age: 34 ± 9 yr; mean BMI: 48.4 ± 8.3 kg/m^2^; waist circumference: 137.0 ± 15.4 cm).

### Laboratory methods

Blood samples were collected from each subject the morning after an overnight fast. Plasma was separated from erythrocytes by centrifugation (15 min, 3500 rpm, 4°C).

Plasma glucose was measured using the hexokinase oxidase method (Beckman Coulter, Galway, Ireland) and plasma insulin levels were assessed using the electrochemiluminescence method (Roche Diagnostic, Mannhein, Germany). Insulin resistance was estimated using the homeostasis model assessment: HOMA-IR = fasting insulin (mUI/L) × fasting glucose (mmol/L)/22.5.

Plasma total cholesterol, HDL-c, LDL-c and triglycerides were determined using enzymatic methods (CHOD-PAP, HDL-c plus and GPOPAP, respectively, Roche, Grenoble, France). Free fatty acids (FFA) were determined using a colorimetric method (Wako Industrials, Osaka, Japan).

Plasma high-sensitivity C-reactive protein (hs-CRP) was measured using the turbidimetric method (Synchron LX^®^, Beckman Coulter, Paris, France).

Serum concentrations of ApoC-II and ApoC-III were determined by an immunoturbidimetric assay using K-ASSAY kits (Kamiya Biomedical Company, Seattle, WA, USA). Serum concentrations of ApoA-I and ApoB-100 were determined by an immunonephelometry assay (Dade Behring Company, Marburg, Germany). Plasma concentrations of ApoB-48 were measured using an ELISA kit (Shibayagi, Gunma, Japan).

Specific ELISA kits were used to measure serum levels of leptin (Active Human Leptin, DSL Systems, Webster, TX, USA), adiponectin (Quantikine Human Adiponectin, R&D Systems, Minneapolis, MN, USA), resistin (Quantikine Human Resistin, R&D Systems, Minneapolis, MN, USA) and ghrelin (Human Unacylated Ghrelin, Biovendor, Montigny, France).

Body composition was assessed by dual energy X-ray absorptiometry (DXA) method (Lunar iDXA and enCORE software version 2007, GE Healthcare, Chalfont St Giles, United Kingdom): body fat mass (BFM) and fat free mass (FFM) compartments were considered. Resting energy expenditure (REE) was measured by indirect calorimetry (Quark-RMR-Cosmed, Rome, Italy).

### Statistical analyses

Statistical analyses were performed using SPSS (Statistical Package for the Social Sciences, version 15.0, Chicago, IL, USA) software. The results are presented as means ± SD. The Mann-Whitney U-test was used for comparisons between groups. Before the analyses, the variables with skewed distributions were logarithmically transformed to normalize their distributions. Correlations were evaluated by linear regression and multiple interactions by stepwise regression and by exhaustive checks of all possible multivariate models. Variables with univariate correlation with a *p *< 0.05 were included in the multivariate regression. The structure of the covariance matrix for each variable was taken into account in all analyses to ensure the most adequate statistical fit and power. A *p*-value < 0.05 was considered statistically significant.

## Results

The anthropometric, body composition, biochemical and hormonal characteristics of the three groups of obese patients and the control group are presented in Table 1. There was no difference in age or gender ratio between the obese groups and the control group.

**Table 1:** Anthropometric, body composition, hormonal and biochemical parameters of obese groups and non-obese control subjects

Variables	MHO (n = 21)	MAO (n = 17)	LHO (n = 21)	Non-obese (n = 21)
Age (yr)	37 ± 5	38 ± 11	34 ± 9	33 ± 6
Gender (Male/female)	4/17	6/11	6/15	5/16
BMI (kg/m^2^)	41.4 ± 4.1	43.3 ± 4.7	48.4 ± 8.3^‡^	22.1 ± 2.9^§,¶^,**
Waist circumference (cm)	121.1 ± 13.5	129.0 ± 14.3	137.0 ± 15.4	74.8 ± 7.6^§,¶^,**
Systolic BP (mmHg)	124.6 ± 5.2	128.5 ± 7.5	127.4 ± 7.1	121.4 ± 5.9
Diastolic BP (mmHg)	78.2 ± 6.4	77.6 ± 9.0	76.8 ± 7.7	74.2 ± 8.9
FFM %	38.0 ± 13.1	33.5 ± 15.2	35.0 ± 14.1	nd
BFM %	43.2 ± 16.4	47.5 ± 12.4	49.8 ± 14.4	nd
REE kcal/d	1608 ± 285	1732 ± 299	1827 ± 352	nd
Glucose (mmol/L)	5.3 ± 0.3	5.4 ± 0.6	5.0 ± 0.5	4.5 ± 0.5^§,¶^
Insulin (mU/L)	16.0 ± 8.3	26.1 ± 10.3*	22.1 ± 7.4	7.1 ± 3.3^§,¶,^**
HOMA-IR score	3.8 ± 2.3	6.1 ± 2.5*	4.5 ± 1.6^†^	1.4 ± 0.7^§,¶,^**
hs-CRP (mg/L)	5.3 ± 2.2	6.5 ± 3.9	13.8 ± 4.6^†,‡^	1.0 ± 0.6^§,¶,^**
Creatinine (μmol/L)	60.6 ± 4.2	65.5 ± 3.8	57.6 ± 5.2	53.7 ± 2.2
Adiponectin (mg/L)	7.5 ± 3.6	4.9 ± 2.9*	6.1 ± 3.4	9.8 ± 4.8^¶,^**
Resistin (ng/mL)	17.9 ± 7.5	17.1 ± 9.0	22.3 ± 13.2	10.9 ± 3.4^§,¶,^**
Leptin (ng/mL)	58.2 ± 22.3	65.3 ± 24.6	82.1 ± 48.4^‡^	12.6 ± 10.3^§,¶,^**
Ghrelin (pg/mL)	185.3 ± 85.3	150.3 ± 61.6	161.2 ± 49	298.6 ± 99.4^§,¶,^**
Free fatty acids (mmol/L)	0.5 ± 0.2	0.6 ± 0.2	0.7 ± 0.2^‡^	0.4 ± 0.2^¶,^**
Triglycerides (mmol/L)	0.9 ± 0.3	2.4 ± 0.8*	1.0 ± 0.2^†^	0.9 ± 0.1^¶^
Total cholesterol (mmol/L)	4.4 ± 0.5	4.4 ± 0.7	3.9 ± 0.7	4.3 ± 0.5
HDL-cholesterol (mmol/L)	1.36 ± 0.1	0.72 ± 0.1*	0.76 ± 0.1^‡^	1.56 ± 0.1^¶,^**
LDL-cholesterol (mmol/L)	3.1 ± 0.5	3.3 ± 0.7	3.1 ± 0.6	2.3 ± 0.5
ApoA-I (g/L)	1.6 ± 0.2	1.28 ± 0.1*	1.2 ± 0.1^‡^	1.7 ± 0.2^¶,^**
ApoB-100 (mg/L)	856.1 ± 165	947.7 ± 289*	771.3 ± 269^†^	736.9 ± 126^¶^
ApoC-III (mg/L)	102.1 ± 20.3	169.5 ± 71.6*	93.8 ± 15.2^†^	114.0 ± 30.1^¶^
ApoC-II (mg/L)	34.5 ± 9.5	56.5 ± 19.5*	29.4 ± 8.4^†^	36.8 ± 10.3^¶^
Total apoC-III ⁄ total apoC-II	3.1 ± 0.7	3.0 ± 0.6	3.2 ± 0.7	2.9 ± 0.6
ApoB-48 (mg/L)	4.1 ± 1.7	8.8 ± 4,0*	3.5 ± 1.7^†^	3.9 ± 1.8^¶^

Values are means ± SD. Statistical significance is from Mann-Whitney U-test.

BFM, body fat mass; BMI, body mass index; BP, blood pressure; FFM, free fat mass; LHO, low HDL-cholesterol obese; MAO, metabolically abnormal obese; MHO, metabolically healthy obese; REE, resting energy expenditure; nd, not determined.

*p < .05 MAO vs. MHO.

^† ^*p *< .05 MAO vs. LHO.

^‡ ^*p *< .05 LHO vs. MHO.

^§ ^*p *< .05 MHO vs. Non-obese.

^¶ ^*p *< .05 MAO vs. Non-obese.

**p < .05 LHO vs. Non-obese.

### Comparisons between the three obese groups and control subjects

As expected, the three obese groups were bigger, had increased waist circumferences, were more insulin resistant and had higher hs-CRP levels than control subjects (p < 0.05 for all parameters). The lipid and apoprotein profiles did not differ between the MHO group and the control group. The MAO group and the LHO groups both had decreased plasma HDL-c and apoprotein A-I levels and increased FFA levels compared to the control group (*p *< 0.05 for all parameters). Only the MAO group with metabolic syndrome showed higher plasma triglycerides and higher levels of apoproteins B-100, B-48, C-II and C-III than the control group (*p *< 0.05 for all parameters). The hormonal profile showed higher serum leptin and resistin levels and lower serum ghrelin levels in the three obese groups compared to the control group (*p *< 0.05 for all parameters), but decreased serum adiponectin levels was only observed in the MAO (*p *< 0.05) and LHO groups (*p *< 0.05) (Table 1).

### Comparisons between MHO and MAO groups

There were no differences in the body composition and the REE between MHO and MAO patients (Table 1).

The lipid profiles showed higher plasma triglycerides, apoproteins B-100, B-48, C-II and C-III and lower HDL-c and apoprotein A-I levels in the MAO group (*p *< 0.05 for all parameters) (Table 1).

The hormonal profile showed higher insulin resistance and lower serum adiponectin levels in the MAO group (*p *< 0.05 for all parameters). However, there was no difference in the levels of serum leptin, resistin and ghrelin (Table 1).

### Comparisons between MHO and LHO groups

The LHO group had a higher BMI than that of MHO patients (*p *< 0.05), but there were no differences in the body composition and REE between the two groups (Table 1).

The lipid profile showed lower HDL-c and apoprotein A-I levels and higher FFA and hs-CRP levels in the LHO patients (*p *< 0.05 for all parameters) (Table 1).

The hormonal profile showed higher serum leptin levels in the LHO group (*p *< 0.05), but there were no difference in insulin resistance or serum adiponectin, resistin or ghrelin levels (Table 1).

### Comparisons between MAO and LHO groups

There were no differences in the body composition and REE between the MAO and LHO groups (Table 1).

The lipid profile showed higher plasma triglycerides, apoproteins B-100, B-48, C-II and C-III levels in the MAO group (*p *< 0.05 for all parameters). Hs-CRP levels were higher in the LHO group (*p *< 0.05) (Table 1).

The hormonal profile showed higher insulin resistance and lower serum leptin levels in the MAO group (*p *< 0.05 for all parameters), but no difference in the serum adiponectin, resistin or ghrelin levels (Table 1).

### Stepwise multivariate analysis

**Figure 1:**
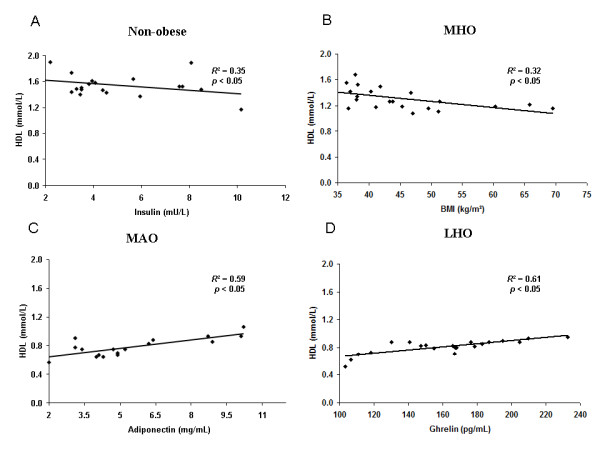
**Association in stepwise regression analysis between plasma HDL-c levels and several parameters in non-obese and obese subjects**. Relationships between HDL-c levels and: plasma insulin in non-obese control subjects (**A)**; body mass index (BMI) in metabolically healthy obese (MHO) patients (**B)**; serum adiponectin in metabolically abnormal obese (MAO) patients (**C)**; serum unacylated ghrelin in isolated low HDL-c obese (LHO) patients (**D)**.

To assess the relationships between HDL-c or triglycerides and several variables, a stepwise multivariate analysis was carried out and adjusted for age, gender, BMI, waist, BFM, FFM, glucose, HOMA-IR, hs-CRP, FFA, apoB-100, apoB-48, apoC-II, apoC-III, apoC-III/apoC-II, insulin, adiponectin, leptin, resistin, and ghrelin for all three groups of obese patients and the non obese control group (Table 2). The analysis showed that, in the control group, insulin was associated negatively and significantly (β = -0.423; *p *= 0.039) with the variability of HDL-c and explained 35% of the plasma HDL-c variance (r^2 ^= 0.35) (Figure 1A). In addition, adiponectin was associated negatively and significantly (β = -0.384; *p *= 0.041) with the variability of triglycerides and explained 22% of the plasma triglycerides variance (r^2 ^= 0.22). In the MHO group, BMI was associated negatively and significantly (β = -0.732; *p *= 0.028) with the variability of HDL-c and explained 32% of the plasma HDL-c variance (r^2 ^= 0.32) (Figure 1B). In addition, adiponectin was associated negatively and significantly (β = -0.414; *p *= 0.037) with the variability of triglycerides and explained 29% of the plasma triglycerides variance (r^2 ^= 0.29). In the MAO group, adiponectin levels contributed positively and significantly (β = 0.52; *p *= 0.045) with the variability of HDL-c and explained 59% of the plasma HDL-c variance (r^2 ^= 0.59) (Figure 1C). In addition, adiponectin was associated negatively and significantly (β = -0.524; *p *= 0.028) with the variability of triglycerides and explained 38% of the plasma triglycerides variance (r^2 ^= 0.38). In the LHO group, unacylated ghrelin levels contributed positively and significantly (β = 0.630; *p *= 0.032) with the variability of HDL-c and explained 61% of the plasma HDL-c variance (r^2 ^= 0.61) (Figure 1D). In addition, leptin was associated negatively and significantly (β = -0.479; *p *= 0.036) with the variability of triglycerides and explained 40% of the triglycerides variance (r^2 ^= 0.40).

**Table 2:** Relationships between plasma HDL-c or triglyceride levels and anthropometric, hormonal and biochemical parameters in obese groups and non-obese control subjects

Variables	*β*	SE	R^2^	*p*-value
**TG levels-non obese**				
**Adiponectin**	-0.384	0.238	0.22	0.041
**FFA**	0.089	0.267	0.22	0.083
**Ghrelin**	0.200	0.163	0.22	0.468
**TG levels-MHO**				
**Adiponectin**	**-**0.414	0.229	0.29	0.037
**HOMA score**	0.247	0.167	0.29	0.116
**Leptin**	0.052	0.005	0.29	0.665
**TG levels-MAO**				
**Adiponectin**	**-**0.524	1.020	0.38	0.028
**HOMA score**	0.371	0.911	0.38	0.064
**FFA**	0.075	0.456	0.38	0.120
**TG levels-LHO**				
**Leptin**	-0.479	0.246	0.40	0.036
**FFA**	-0.331	0.197	0.40	0.062
**Resistin**	-0.189	0.005	0.40	0.678
**HDL-c levels-non obese**				
**Insulin**	-0.423	0.219	0.350	0.039
**Leptin**	-0.130	0.159	0.350	0.090
**Ghrelin**	-0.056	0.045	0.350	0.103
**HDL-c levels-MHO**				
**BMI**	-0.732	0.345	0.320	0.028
**Insulin**	-0.120	0.102	0.320	0.097
**adiponectin**	0.113	0.069	0.320	0.234
**HDL-c levels-MAO**				
**Adiponectin**	0.520	2.569	0.59	0.045
**Ghrelin**	0.390	0.234	0.59	0.148
**Insulin**	0.012	0.671	0.59	0.472
**HDL-c levels- LHO**				
**Ghrelin**	0.630	1.478	0.61	0.032
**Leptin**	0.249	0.315	0.61	0.129
**Insulin**	0.035	0.061	0.61	0.158

Multivariate regression models for the relationships between plasma HDL-c or triglyceride levels and age, gender, body mass index (BMI), waist, body fat mass (BFM), fat free mass (FFM), glucose, homeostasis model assessment-insulin resistance (HOMA-IR), hs-C-reactive protein (hs-CRP), free fatty acids (FFA), apoA-I, apoB-100, apoB-48, apoC-II, apoC-III, apoC-III/apoC-II, insulin, adiponectin, leptin, resistin, ghrelin for obese and non-obese groups.

## Discussion

In this study, we have shown that specific hormone profiles were associated with HDL-c levels in the three subsets of obese patients we investigated: metabolically healthy obese (MHO) patients, metabolically abnormal obese (MAO) patients and isolated low-HDL-c obese (LHO) patients. Our main results show a strong and positive correlation between HDL-c and unacylated ghrelin levels in the LHO group, and between HDL-c and adiponectin levels in the MAO group, independently of insulin resistance and hs-CRP levels in both cases.

The first subset of our obese patients corresponded to MHO patients described in the literature, which is thought to represent between 20-30% of the obese population [6]. Their lipid and apoprotein profiles were indistinguishable from our non-obese control group. Despite the difference in insulin resistance between MHO and control groups, we confirmed the high insulin sensitivity of this group compared to MAO and LHO obese groups [6]. High adiponectin concentrations have been associated with the MHO phenotype [6]. We also documented higher serum adiponectin levels in our MHO group compared to the MAO group, but these two groups displayed the same hormonal profiles regarding adipose tissue-derived leptin or resistin and stomach derived ghrelin.

The second subset corresponded to the MAO patients, a group that displayed the typical dyslipidaemia seen in insulin resistant patients. This pathophysiology is widely explained by the accumulation of triglyceride-rich lipoproteins (TRL) from the liver (TRL-apoB-100 or VLDL) and from the intestine (TRL-apoB-48 or chylomicrons). This accumulation of TRL has been attributed to the overproduction of VLDL [3] and chylomicrons [15] associated with a defective TRL removal due to the reduction of lipoprotein lipase (LPL) activity mainly in the post-prandial state [16] in a context of competition between VLDL and chylomicrons for this common, saturable, removal pathways, to abnormalities in the apoprotein composition of TRL [17] and to a defect in the hepatic uptake of TRL [16]. The increase in the plasma resident time of the TRL would enhance cholesterol ester transfer protein (CETP) exchange of triglycerides and cholesteryl ester between HDL, and LDL on one hand and TRL on the other hand leading to the increase of HDL catabolism and the increase formation of small and dense LDL [3,16]. In this study, we have confirmed that a high level of insulin resistance is a driving parameter in the pathophysiology of the dyslipidaemia seen in the MAO group compared to the MOA and LHO groups and found the typical lipid profile: increased plasma triglycerides, apoB-100, apoB-48, apoC-III and apoC-II, and decreased HDL-c and apoA-I. The same total apoC-III/apoC-II ratios between the subgroups of obese patients suggest that this parallel increase in apoproteins having opposite functional effects in the TRL clearing process is most likely secondary to the increase of plasma TRL, as we have previously reported for type 2 diabetic patients [18]. In our multiple regression analysis, adiponectin levels were strongly and positively correlated with the HDL-c levels and were negatively correlated with TG levels independently of BMI, insulin resistance and hs-CRP levels. In our study, this negative association between adiponectin and TG levels was shared by the control and the MHO and MAO groups but not by the LHO group. This difference could be caused by the complexity of the factors regulating TG metabolism. Indeed, a kinetic study has shown that adiponectin, but not leptin or resistin, was the most significant predictor for plasma VLDL apoB concentration, independently of both insulin resistance and size of adipose tissue compartments in subjects with a large range of BMI (from 22 to 35 kg/m^2^) but that plasma VLDL apoB kinetic was controlled differently, with adiponectin and total body fat regulating catabolism and insulin resistance regulating hepatic secretion [19]. Adiponectin levels explained 59% of the variance of HDL-c, thus showing a tight link between these two parameters. A positive correlation between plasma adiponectin and HDL-c has already been found in several studies that have included non diabetic [20], diabetic [21] and obese patients [22]. Moreover, two kinetic studies have found similar results showing a strong negative correlation between adiponectin and apoA-I FCR (fractional catabolic rate) [22,23], although in one study, this only occurred in the non-obese group [22]. This negative correlation has been shown to be independent of BMI, body fat distribution and insulin sensitivity, which suggests that adiponectin may have a direct role in HDL metabolism [23]. The inverse relationship shown in vivo between adiponectin and plasma hepatic lipase activity in non diabetic and diabetic patients could be a link between plasma adiponectin levels and HDL metabolism [24].

The third subset of our obese patients, characterised by isolated low-HDL-c levels (LHO) represented an intermediate obese population between the MHO and MAO groups. Despite a higher BMI that explained the significantly higher levels of serum leptin, the insulin sensitivity of this group is in between the two other groups. A positive association has been shown between BMI and triglyceride concentration, which is stronger for visceral than for subcutaneous abdominal adipose tissue [25], but we did not assess these two parameters. Despite higher BMI, LHO patients displayed normal triglyceride levels. Their higher BMI could reflect a better expandability of adipose tissue in the LHO group, permitting a safer storage of fat and avoiding metabolic syndrome [26]. Indeed, the lipid profile of LHO group was comparable to that of the MHO group, with the exception of FFA, HDL-c and apoA-I levels. We only found a negative correlation between leptin and triglyceride levels in the LHO group. Their normal triglyceride concentrations could be due to a better insulin and leptin sensitivity in this group compared to the MAO group. Indeed, leptin has been negatively associated with hepatic VLDL-TG secretion rate independently of plasma FFA concentrations, possibly by stimulating hepatic fatty acid oxidation and decreasing *de novo *lipogenesis [27]. Hs-CRP levels were significantly higher in the LHO group compared to the two other obese groups and could partly explain the low HDL-c levels. Indeed, the metabolism, plasma level and apolipoprotein contents of HDL can be strongly impaired in acute and chronic inflammation, including obesity [28]. Nevertheless, multiple regression analysis showed that only unacylated ghrelin levels were positively correlated with HDL-c levels, which explained 61% of the variance of HDL-c, thus demonstrating a tight link between these two parameters. The association between plasma ghrelin levels and insulin resistance is debated. Whereas low levels of total plasma ghrelin have been associated with insulin resistance [29], the same negative association has been shown between plasma total or deacylated ghrelin and insulin resistance but a positive association between plasma acylated ghrelin or acylated to deacylated ghrelin ratio and insulin resistance [13]. In the present study, the significant association between low serum unacylated ghrelin (we did not measure serum acylated ghrelin for technical reasons) and low HDL-c levels could be partly explained by the same pathophysiology of the MAO group. However, our LHO group did not display hypertriglyceridaemia. An explanation for this could be an increased in post-prandial response in triglyceride levels that was not seen in our fasting measurements. Alternatively, serum ghrelin could act more directly in HDL metabolism by interacting with a species of HDL associated with paraoxonase and apolipoprotein J [30], but further studies are needed to characterize this mechanism. Several HDL turnover studies have been performed to elucidate the mechanism that results in low HDL-c levels, and have included subjects with normal TG levels, but the results are contradictory. On the one hand, Le *et al *found decreased apoA-I production rate and normal apoA-I FCR in low-HDL-c and normal TG subjects compared to normal HDL-c and normal TG subjects [31]. On the other hand, Brinton *et al *and Gylling H *et al *found increased apoA-I FCR in low-HDL-c and normal TG subjects [32,33] (as it has been shown in these and other studies in low-HDL-c and high TG subjects [32,34]), but no difference in apoA-I production rate compared to normal HDL-c and normal TG subjects [32,33]. HDL-c levels are under considerable genetic control with heritability estimates of up to 80% [12]. Among the genetic variants of genes involved in HDL metabolism, a polymorphism (g.-1062 G > C) identified in the promoter region of the ghrelin gene has been independently associated with serum HDL-c levels in a population of type 2 diabetic Korean patients [35]. However, another study found no association between five SNPs in the ghrelin gene and HDL-c levels in a Canadian population [36]. Further genetic studies are needed.

In conclusion, this study shows that the HDL-c levels are associated with a specific plasma hormonal profile in different subsets of class II and III obese patients. The low HDL-c level in the metabolically abnormal obese (MAO) patients was mainly driven by insulin resistance and was associated with a low level of serum adiponectin. The isolated low-HDL-c obese (LHO) patients represented a very interesting population with a level of insulin sensitivity that was between those of the MAO group and the metabolically healthy obese (MHO) group. These patients also showed a high level of hs-CRP and both of these factors could partly explain the low HDL-c levels associated with a direct or indirect role of serum ghrelin levels. Further studies are needed to investigate the direct or indirect role of ghrelin and adiponectin, and to better assess the importance of the genetic background or hormonal profile in HDL-c metabolism of obese patients.

## Abbreviations

Apo: Apoprotein; BFM: Body fat mass; BMI: Body mass index; DXA: Dual energy X-ray absorptiometry; FCR: Fractional catabolic rate; FFA: Free fatty acids; FFM: Fat free mass; HDL-c: High-density lipoprotein-cholesterol; HOMA-IR: Homeostasis model assessment-insulin resistance; hs-CRP: High-sensitivity C-reactive protein; LDL-c: Low-density lipoprotein-cholesterol; LHO: Low HDL-c obese; MAO: Metabolically abnormal obese; MHO: Metabolically healthy obese; REE: Resting energy expenditure; TG: Triglycerides; TRL: Triglyceride-rich lipoproteins; VLDL: Very low-density lipoprotein.

## Competing interests

The authors declare that they have no competing interests.

## Authors' contributions

JPN, MM, RV: conception and design, data collection, data analysis, manuscript writing. SB, AML, BB, ABL, ND, RG, CM, AN, HP, BV: data collection, data analysis. JG: statistical analysis. All authors read and approved the final manuscript.
